# Functional divergence of diacylglycerol acyltransferases in the unicellular green alga *Haematococcus pluvialis*

**DOI:** 10.1093/jxb/eraa451

**Published:** 2020-10-01

**Authors:** Haiyan Ma, Xiaoying Wu, Ziwang Wei, Liang Zhao, Zhongze Li, Qing Liang, Jie Zheng, Yu Wang, Yanhua Li, Linfei Huang, Qiang Hu, Danxiang Han

**Affiliations:** 1 Center for Microalgal Biotechnology and Biofuels, Institute of Hydrobiology, Chinese Academy of Sciences, Wuhan, China; 2 Laboratory for Marine Biology and Biotechnology, Qingdao National Laboratory for Marine Science and Technology, Qingdao, China; 3 Institute for Advanced Study, Shenzhen University, Shenzhen, China; 4 Key Laboratory for Algal Biology, Institute of Hydrobiology, Chinese Academy of Sciences, Wuhan, China; 5 University of Chinese Academy of Sciences, Beijing, China; 6 The Innovative Academy of Seed Design, Chinese Academy of Sciences, Beijing, China; 7 Beijing Key Laboratory of Algae Biomass, SDIC Biotech Investment Corporation, Beijing, China; 8 Department of Chemistry, The University of British Columbia, Vancouver, BC, Canada; 9 University of Exeter, UK

**Keywords:** Acyl-CoA:diacylglycerol acyltransferase (DGAT), functional divergence and convergence, lipids, lysophosphatidic acyltransferase (LPAAT), microalgae, triacylglycerol biosynthesis

## Abstract

Acyl-CoA:diacylglycerol acyltransferase (DGAT) catalyzes the final committed step in triacylglycerol biosynthesis in eukaryotes. In microalgae, the copy number of DGAT genes is extraordinarily expanded, yet the functions of many DGATs remain largely unknown. This study revealed that microalgal DGAT can function as a lysophosphatidic acyltransferase (LPAAT) both *in vitro* and *in vivo* while losing its original function as DGAT. Among the five DGAT-encoding genes identified and cloned from the green microalga *Haematococcus pluvialis*, four encoded HpDGATs that showed triacylglycerol synthase activities in yeast functional complementation analyses; the exception was one of the type II DGAT encoding genes, *HpDGTT2*. The hydrophobic recombinant HpDGTT2 protein was purified in soluble form and was found to function as a LPAAT via enzymatic assay. Introducing this gene into the green microalga *Chlamydomonas reinhardtii* led to retarded cellular growth, enlarged cell size, and enhanced triacylglycerol accumulation, identical to the phenotypes of transgenic strains overexpressing CrLPAAT. This study provides a framework for dissecting uncharacterized DGATs, and could pave the way to decrypting the structure–function relationship of this large group of enzymes that are critical to lipid biosynthesis.

## Teaser

One copy of acyl-CoA:diacylglycerol acyltransferase (DGAT) from microalgae can function as a lysophosphatidic acyltransferase both *in vitro* and *in vivo* while losing its original function as DGAT.

## Introduction

Triacylglycerols (TAGs) are the major storage lipids in most eukaryotes, including plants, algae, fungi, and animals. In plants and algae, photosynthetically reduced carbons are used for *de novo* fatty acid biosynthesis in the plastids; the fatty acids are then assembled with a glycerol backbone via sequential acylation reactions in the *de novo* TAG biosynthesis pathway to form TAGs (Hu *et al.*, 2008). As well as functioning as an energy reservoir and as signaling molecules, TAGs are involved in many biological processes in photosynthetic organisms, such as the development of seeds and pollen, senescence of leaves, and protection of photosystems under stress conditions such as high light and nitrogen starvation (Yang and Benning, 2018).

The final committed step for TAG biosynthesis in the Kenney pathway is catalyzed by acyl-CoA:diacylglycerol acyltransferases (DGATs; EC 2.3.1.20), which are mainly encoded by two distinct gene families, referred to as *DGAT1* and *DGAT2* (Ohlrogge and Jaworski, 1997; Lung and Weselake, 2006). Both DGAT1 and DGAT2 are membrane-bound proteins primarily associated with the endoplasmic reticulum, most likely localized in different subdomains (Shockey *et al.*, 2006). Despite the fact that a few other enzymes possess DGAT activity, including a soluble-type DGAT3 reported in *Arachis hypogaea* (Saha *et al.*, 2006) and a bifunctional wax synthase/DGAT in *Arabidopsis thaliana* (Li *et al.*, 2008), DGAT1 and DGAT2 have received tremendous attention due to their evolutionary conservation across most plants and algae, as well as their biotechnological significance for increasing the oil production of seeds and oleaginous microalgae (Weselake *et al.*, 2009; Liang and Jiang, 2013; Bellou *et al.*, 2014).

The *DGAT2* gene family was found to be extensively expanded in microalgal genomes (Chen and Smith, 2012). In the model green alga *Chlamydomonas reinhardtii*, there are five copies of the *DGAT2* gene (Boyle *et al.*, 2012). The heterokont microalga *Nannochloropsis oceanica* possesses 11 *DGAT2* genes, and this is believed to relate to its oleaginous traits (Vieler *et al.*, 2012; Li *et al.*, 2014; Wang *et al.*, 2014). Eight copies of *DGAT2* isoforms were recently cloned and characterized in *Chlorella zofingiensis* (Mao *et al.*, 2019). Nevertheless, many *DGAT2* genes do not show any response to environmental stresses at the transcript level, while algal cells accumulate enormous amounts of TAGs (Boyle *et al.*, 2012; Li *et al.*, 2014; Mao *et al.*, 2019). For *C. reinhardtii*, among the five annotated *DGAT2* genes, *DGTT1* was the only one found to be responsive to nitrogen depletion. The levels of transcripts of the four other *DGAT2* genes (*DGTT2*, *3*, *4*, and *5*) remained almost constant over time or at a very low abundance (Boyle *et al.*, 2012). For *N. oceanica*, six of eleven *DGAT2* isoforms were up-regulated under N-depleted conditions, whereas four copies were up-regulated under N-replete conditions, and one copy was non-responsive to nitrogen availability (Li *et al.*, 2014). In *C. zofingiensis*, four of eight *DGAT2* genes were up-regulated under nitrogen deficiency (Mao *et al.*, 2019). Moreover, many algal *DGAT2* isoforms could not complement the TAG-deficient phenotype of *Saccharomyces cerevisiae* H1246, a tool frequently used to verify the function of DGAT, nor could they exhibit any enzymatic activity *in vitro* (Liu *et al.*, 2016; Xin *et al.*, 2017; Mao *et al.*, 2019; Xin *et al.*, 2019). These findings raise a crucial question about the authentic functions of these cryptic *DGAT2* genes in microalgae. Functional divergence may have occurred in the microalgal *DGAT2* gene family during evolution.

The green microalga *Haematococcus pluvialis* is well acknowledged for its outstanding capability in synthesizing astaxanthin (3, 3′-dihydroxy-β, β-carotene-4,4′-dione), a natural ketocarotenoid with strong antioxidant activity (Boussiba, 2000; Han *et al.*, 2013). *Haematococcus pluvialis* accumulates astaxanthin in the form of mono- and di-esters under various stress conditions, such as high light, nutrient (e.g. nitrogen) deprivation, and high salinity. Astaxanthin esters are stored in lipid bodies (LBs) that consist of the bulk of TAGs and sterol esters, which are assumed to serve as a matrix for solubilizing astaxanthin esters (Lee and Zhang, 1999; Boussiba, 2000). The capabilities of *H. pluvialis* to orchestrate the production of a broad spectrum of lipids mean that it has great potential as a biodiesel feedstock (Damiani *et al.*, 2010). In *H. pluvialis*, DGATs are not only the committed enzymes for TAG biosynthesis, but have also been deduced to be the enzymes catalyzing the formation of astaxanthin esters (Chen *et al.*, 2015). Thus, understanding the function of HpDGATs will aid in the genetic engineering of *H. pluvialis* for the production of economically viable biodiesels along with high-value bioproducts. One homolog of *DGAT1* and four *DGAT2* genes have been identified in the *Haematococcus* transcriptome database (Ma *et al.*, 2018). However, their functions remain largely uncharacterized.

In this study, five *HpDGAT*s were cloned and characterized with the aim of identifying *DGAT* genes with novel function(s) and thereby to understand the functional divergence of the gene family. Since the first report of DGAT activity in the 1950s, advances in understanding their structure–function relationship have remained limited due to the hydrophobicity of these enzymes and difficulties in purifying them (Liu *et al.*, 2012). In this study, we successfully purified a highly hydrophobic recombinant HpDGAT2 in soluble form, which was found to function as a lysophosphatidic acid acyltransferase (LPAAT) that utilizes lysophosphatidic acid (LPA) and a broad spectrum of acyl-CoA as substrates to produce phosphatidic acid (PA) *in vitro*. In contrast to DGAT, which catalyzes the final committed acylation step, LPAAT catalyzes the second acylation step and adds an acyl group to the *sn*-2 position of the glycerol backbone in the *de novo* TAG biosynthesis pathway. Overexpression of this *HpDGAT2* gene could not restore the TAG-deficient phenotype of *S. cerevisiae* H1246. Nevertheless, introducing this gene into *C. reinhardtii* led to retarded cellular growth, enlarged cell size, and enhanced TAG accumulation, phenotypes that are identical to the phenotypes of transgenic strains overexpressing CrLPAAT1, a well-studied plastidial LPAAT-encoding gene originally from *C. reinhardtii* (Yamaoka *et al.*, 2016). This study provides a framework for dissecting the functions of uncharacterized DGATs in microalgae and other organisms, and broadens our understanding of the functional divergence of this gene family.

## Materials and methods

### Strain and culture conditions

*Haematococcus pluvialis* NIES144 was obtained from the National Institute for Environmental Studies, Tsukuba, Japan. Algal cells were cultured in 100 ml of basal medium (Kobayashi *et al.*, 1991) in a 250 ml Erlenmeyer flask at 20 °C and under irradiance of 20 μmol m^−2^ s^−1^ (low light; LL) with a 12 h/12 h light/dark cycle, which are the normal conditions for growing *H. pluvialis* cells. After 4 days of cultivation, the cell density reached ~3×10^5^ cells ml^−1^, and the algal cells were then exposed to continuous illumination of 200 μmol m^−2^ s^−1^ (high light; HL) and grown for 72 h. The pH was maintained at 7.0–7.6 with 40 mM HEPES buffer (Sigma-Aldrich, USA). The LL conditions were used as the control condition when the effects of HL stresses were investigated.

### RNA preparation and RNA-seq

Algal cells cultivated under LL (at day 4) and HL (at 3, 6, 12, 24, 48, and 72 h) were harvested by centrifuging at 1500 *g* for 10 min. The cell pellets were frozen at –80 °C until use. Total RNAs were extracted by resuspending the algal pellets in 1 ml of Trizol reagent® (Invitrogen, USA) in a 2 ml RNase-free tube containing 200 μl of glass beads (Sigma-Aldrich, USA). After disruption with a Minibeadbeater (2.5×10^3^ oscillations per minute for 15 s, applied twice), the cell homogenates were placed on ice for 5 min and then centrifuged at 13 000 *g* for 10 min at 4 °C. The supernatant was carefully transferred to a new 1.5 ml RNase-free tube, and 200 μl of chloroform was added. After vigorously shaking for 15 s, the tube was placed on ice for 2 min and then centrifuged at 13 000 *g* for 15 min at 4 °C. The aqueous phase (~900 μl) was carefully removed to a new RNase-free centrifuge tube without disturbing the interface and then mixed well with isopropanol (half the volume of the aqueous phase). The precipitated pellets were washed with 1 ml of 75% (w/w) ice-cold ethanol, and then centrifuged at 7500 *g* for 5 min at 4 °C. The supernantant was carefully removed, and the pellets were dried at room temperature for 10 min before being dissolved in 40–50 μl of diethyl pyrocarbonate-treated deionzied water. To prepare the library for sequencing, mRNAs were purified by using NEBNext Poly(A) mRNA Magnetic Isolation Module (New England Biolabs, USA). For each time point, three biological replicates were prepared.

Directional transcriptome libraries were prepared by using NEBNext Ultra Directional RNA Library Prep Kit for Illumina (New England Biolabs, USA), which adopts the dUTP method (Parkhomchuk *et al.*, 2009) for strand specificity. The library was sequenced for 2×150 bp runs (paired-end) using an Illumina Hiseq 2500 system. To ensure quality, adapter pollution and low-quality reads were deleted with Trimmomatic (version 0.35) (Bolger *et al.*, 2014). The filtered reads were assembled by using the Trinity platform (25 k-mers, version 2.5.1) for *de novo* transcriptome assembly without genome reference (Grabherr *et al.*, 2011). For each of the mRNA-seq datasets, gene expression was measured as the numbers of aligned reads to the transcript assembly by running the alignment-free method Salmon (version 0.8.1) (Patro *et al.*, 2017), which provided transcript-level estimates of the count of RNA-seq fragments and normalized expression metrics as transcripts per million transcripts (TPM). Differentially expressed genes were identified by using the Bioconductor package DESeq2. Genes were considered to be significantly differentially expressed if the following criteria were met: their expression values showed at least a 2-fold change with a false discovery rate (FDR)-corrected *P*-value ≤0.05 between the control and stress conditions, and their counts-per-million (CPM) values under each condition were ≥5. Gene ontology (GO) enrichment analysis was performed at http://www.omicshare.com/tools/Home/Soft/gogsea.

The longest transcripts of given putative genes were identified as unigenes, and the corresponding protein sequences were subjected to BLAST searches in the databases Uniprot (https://www.uniprot.org/uniprot/) and PFAM (http://pfam.xfam.org/) for functional annotation. The putative acyltransferases of *H. pluvialis* were identified by searches using the keyword “acyltransferase”. The putative *DGAT1* and *DGAT2* genes were identified by BLAST searches of the local transcript database with the *DGAT1* gene of *A. thaliana* (*AtDGAT1*, accession number NP_179535) and *DGAT2* genes from *C. reinhardtii* (*CrDGTT*s, accession numbers AGO32156, AGO32157, AGO32158, and AGO32159) as query sequences.

Validation of the RNA-seq results was performed by reverse transcription–quantitative PCR (RT–qPCR) techniques. *H. pluvialis* NIES144 was cultured as described above, and 5 ml of cell culture was taken for RNA extraction at 12 and 24 h after exposure to HL stress. After centrifugation at 1000 *g* for 5 min at 25 °C, the pellets were frozen in liquid nitrogen, and stored at –80 °C before use. Cell culture under the LL condition was used as a control for gene expression analysis.

RNA was extracted as described above. The PrimeScript^TM^ RT reagent Kit with gDNA Eraser (Perfect Real Time, Takara, Japan) was used for the first-strand cDNA synthesis. The RNA was treated with gDNA Eraser for 5 min at 42 °C, and then reverse transcribed to cDNA with random hexamer primers following the manufacturer’s instructions. The resulting products were diluted 10-fold, and then were used as the template for qPCR. The 18s rRNA gene was used as an internal reference. The primers use for amplifying the five *HpDGAT* genes are listed in Supplementary Table S1 at *JXB* online. The relative fold change in expression was calculated using the 2^−ΔΔCt^ method (Livak and Schmittgen, 2001).

### Cloning and analysis of *DGAT* genes

Algal cells cultivated under LL (at day 4) and HL (at 3, 6, 12, 24, and 48 h) conditions were pooled for total RNA extraction and cDNA library construction. A full-length cDNA pool was synthesized by using the SMART™ cDNA Library Construction Kit (Clontech, Takara, Japan). The double-stranded cDNA was synthesized by using the primer extension method with 5′ PCR Primer and CDS III/3′ PCR Primer. The resulting PCR product was used as a full-length cDNA template for rapid amplification of cDNA ends (RACE). Primers used for RACE were designed at using Primer-BLAST (https://www.ncbi.nlm.nih.gov/tools/primer-blast/) and the primer sequences are listed in Supplementary Table S1. PCR was performed with the Advantage 2 PCR kit (Clontech, Takara, Japan) by using a touchdown approach. Full-length cDNA coding *HpDGAT*s were amplified from the cDNA library, and the PCR products were sequenced for verification.

Each of the five HpDGAT amino acid (AA) sequences obtained was used as a query sequence for BLAST searches at https://blast.ncbi.nlm.nih.gov/Blast.cgi. We selected 18 homologs of HpDGAT1 and 29 homologs of HpDGTTs from eudicot plants (*A. thaliana*, *Brassica napus*), mammals (*Homo sapiens*, *Mus musculus*, *Rattus norvegicus*), amphibians (*Xenopus tropicalis*), fish (*Danio rerio*), birds (*Gallus gallus*), invertebrates (*Drosophila melanogaster*, *Apis cerana*), fungi (*Aspergillus niger*), yeast (*Saccharomyces cerevisiae*), and several species of algae for phylogenetic tree construction. Detailed information on the homologs is listed in Supplementary Table S2. The AA sequences were aligned with ClustalW and the phylogenetic tree was constructed by using the maximum likelihood method based on the JTT matrix-based model (MEGA 7.0 software). For visualization of the motifs in the DGAT homologs, sequence alignments of the DGAT1s and DGAT2s were performed by using Lasergene software (DNAStar, USA). The transmembrane domains of all five HpDGATs were predicted using TMHMM (http://www.cbs.dtu.dk/services/TMHMM/), TMpred (https://embnet.vital-it.ch/software/TMPRED_form.html), and HMMTOP (http://www.enzim.hu/hmmtop/index.php).

### Heterologous expression of *DGAT* genes in *S. cerevisiae* H1246

*Saccharomyces cerevisiae* H1246 (Tetra Mut. relevant genotype: *MAT*α *are1*-Δ::*HIS3 are2*-Δ::*LEU2 dga1*-Δ::*KanMX4 Iro1*-Δ::*TRP1 ADE2 ura3* TAG^−^ SE^−^), which contains knockouts of *DGA1*, *LRO1*, *ARE1*, and *ARE2* (four genes contributing to TAG synthesis), was used to investigate the function of the HpDGATs. Competent *S. cerevisiae* cells were prepared by following the protocol of the *S.c.* Easy Comp^TM^ Transformation Kit (Invitrogen, USA). The primers used for constructing pYES2-DGAT vectors are listed in Supplementary Table S1. Transformation of competent *S. cerevisiae* H1246 cells was performed according to the manufacturer’s instructions. The expression of recombinant HpDGATs was checked by western blotting. The cell pellet collected after induction was resuspended in 500 μl of lysis buffer (0.1 M Tris–HCl, pH 7.4), and the cells were disrupted once by using a Minibeadbeater as described above. The homogenized sample was centrifuged at 1500 *g* for 5 min at 4 °C, and the supernatant was transferred to a new 1.5 ml centrifuge tube and centrifuged at 20 216 *g* for 45 min at 4 °C. The membrane protein fractions for western blotting were prepared as described in Chen *et al* (2015).

For the fatty acid feeding assays, 90 μM of fatty acid buffer containing 1 g l^−1^ bovine serum albumin (BSA; fatty acid free) was added to 10 ml of yeast culture. After 24 h, the yeast cells were stained with 10 μM of BODIPY 493/503 (Invitrogen, USA) and observed under a confocal laser scanning microscope (DLS, Leica, Germany) to examine the formation of lipid bodies (LBs). The lipids were extracted as described in Yoon *et al* (2012) and checked by thin layer chromatography (TLC) developed via petroleum ether/diethyl ether/acetic acid (80:20:1) elution. The lipid spots were visualized by heating at 140 °C after spraying with 10% (w/v) CuSO_4_ in 8% (w/w) H_3_PO_4_ buffer. The C18:1/C18:1/C18:1 TAG (Sigma-Aldrich) was introduced as a standard.

### TAG synthesis in *in vitro* assay

Microsomes of the yeast transformants were prepared as described in Liu *et al* (2016), and were used as enzymatic sources in an *in vitro* assay. The *in vitro* assay was performed in a volume of 200 μl containing the following components: 100 μg of microsomal proteins, 0.1 M Tris–HCl (pH 7.4), 0.125% (w/v) BSA, 200 μM acyl-CoA, and 200 μM C16:0/C16:0 and C18:1/C18:1 diacylglycerol (DAG). The reaction was performed at 30 °C for 1 h with 1200 rpm agitation. The lipids were immediately extracted from the aqueous reaction system with 500 μl of extraction buffer (chloroform:methanol:12 M HCl, 40:40:0.26, v/v/v), and the organic phase after centrifugation was dried under a vacuum concentrator. After drying, the lipid extracts were recovered in either chloroform or methanol:dichloromethane (3:1) for TLC of neutral lipids, as described above.

### Expression and purification of pMAL-DGTT2

The *DGTT2* gene was subcloned into the plasmid pMAL-c5x (NEB, USA) with the primers listed in Supplementary Table S1. *Escherichia coli* BL21 cells were transformed with the constructed plasmid. Clones transferred with the empty pMAL-c5x plasmid were as a control. A single colony was inoculated into 10 ml of Luria–Bertani medium with ampicillin (final concentration100 μg ml^−1^) and cultured overnight at 37 °C on an orbital shaker at a speed of 220 rpm. A 200 ml volume of autoinduction ZYP-5052 medium (Studier, 2005) was inoculated with 1 ml of the overnight culture and then cultured at 37 °C for 5.5–6 h. When the OD_600_ reached ~2, the cells were cultured at 26 °C overnight with the addition of ampicillin (final concentration100 μg ml^−1^) to keep the stability of the transformed plasmid. The *E. coli* cells were harvested by centrifugation at 4000 *g* for 10 min at 4 °C. Purification of the recombinant protein was performed with amylose resin according to the manufacturer’s instructions (NEB, USA). Briefly, the pellets were resuspended in 25 ml of column buffer containing 1 mM DTT (BBI Life Science, China) and proteinase inhibitor (cOmplete Tablets, Roche, Switzerland). The cells were ruptured by using a high-pressure homogenizer at 1000 bar. The homogenate was centrifuged at 20 000 g for 40 min at 4 °C. The supernatant was mixed well with amylase resin overnight at 4 °C with rotation at low speed. The mixture was loaded on to a gravity column and then washed with 20 ml of column buffer. The target protein was eluted by 20 ml of column buffer with 20 mM maltose. The expression and purity of the protein were checked after separation on SDS-PAGE gel. The protein fractions were concentrated, quantified by using a CB-X^TM^ Protein Assay kit (Bioscience, USA), and stored at –80 °C before use.

### *In vitro* enzymatic assay with the recombinant DGTT2

The 200 μl enzymatic assay system contained 100 μg of purified recombinant DGTT2 protein, 0.1 M Tris–HCl (pH 7.4), 0.125% (w/v) BSA, 200 μM acyl-CoA, and 200 μM acyl acceptors [DAG, monoacylglycerol (MAG), LPA-C18:1, or glycerol-3-phosphate (G-3-P)]. The reaction was performed at 30 °C with 1200 rpm agitation for 1 h. The lipid was extracted with the extraction buffer described above. The lipid extracts were recovered in chloroform:methanol (2:1, v/v) and were further separated on a TLC plate with neutral or polar developing solvent chloroform:methanol:H_2_O (65:25:4, v/v/v). The produced PA spot, visualized via iodine vapor staining, was scraped from the plate for structural confirmation by using liquid chromatography-mass spectrometry (LC-MS). The identity of PA was verified with parent scanning of *m*/*z* 153, and the acyl groups of PA were resolved as acyl anions [RxCO_2_]^−^ fragmented from the daughter scan (Welti *et al.*, 2002).

For substrate preference analysis, 10 types of acyl-CoA species (Avanti, USA), 14:0, 16:0, 16:1, 18:0, 18:1, 18:2, 18:3(n-3), 18:3(n-6), 20:4, and 20:5, were used as acyl donors, and LPA-C18:1 (Avanti, USA) was used as the acyl acceptor. The effects of Mg^2+^ and Mn^2+^ on enzymatic activities were determined at concentrations ranging from 0 to 20 mM. A reaction without the addition of LPA was used as the negative control. The PA was quantified through indirect measurement of the free thiolates (HSCoA) produced in the reaction with 5,5′-dithiobis (2-nitrobenzoic acid) (DNTB) (Ellman, 1959). The HSCoA released from the acyl-CoA reacted with DNTB, producing 2-nitro-5-thiobenzoic acid, which has a maximum absorption peak at 412 nm. Cysteine at concentrations of 0–200 μM was applied to establish the standard curve for quantification.

### Transformation of *C. reinhardtii* with *HpDGTT2* and *CrLPAAT1*

The cDNAs encoding *HpDGTT2* and *CrLPAAT1* were subcloned into the vector pClamy_4 (Invitrogen, USA) with the primers listed in Supplementary Table S1. *Chlamydomonas reinhardtii* CC-400 cw15 cells were transformed with *Sca*I-linearized vectors. After incubation at 26 °C for 6–8 days, the colonies that appeared on agar plates containing Tris-acetate-phosphate medium with zeocin (10 μg ml^−1^) were selected, and the insertion of the *HpDGTT2* or *CrLPAAT1* gene into the genome of *C. reinhardtii* was checked by PCR with the universal primer set FP1 (5′-TCGTGTCCACGAACTTCCG-3′) and SRP1 (5′-ATTTACCTCCGCGAGCAACT-3′). The expression of *HpDGTT2* and *CrLPAAT1* in the positive clones was quantified by RT–qPCR. The *CBLP* gene (Schloss, 1990) was used as an internal reference. The primers for amplifying each gene are listed in Supplementary Table S1. The relative expression of *HpDGTT2* and *CrLPAAT1* gene determined using the 2^−ΔCt^ method.

For each foreign gene, two of the positive clones were selected and were inoculated into 10 ml of the TAP growth medium. After growing for about 1 week under a light density of 30 μmol m^−2^ s^−1^ at 25 °C on an orbital shaker at a speed of 120 rpm, the cell culture was inoculated into 200 ml of TAP growth medium in a 500 ml Erlenmyer flask with an initial cell concentration of 2.5×10^5^ ml^−1^. After 3 days, the cell cultures were transferred to TAP growth medium without nitrogen and were grown for a further 3 days. The cell growth was monitored on a daily basis. The algal cells were collected, stained with BODIPY (final concentration 10 μM) for 10 min, and analyzed by flow cytometry (Cytomics FC500, Beckman Coulter, Germany) for cell size measurement (channel FSC). LBs were visualized under fluorescence microscopy (BX53, Olympus, Japan). For detecting the level of reactive oxygen species (ROS), the algal cells were stained with 10 μM 2′,7′-dichlorodihydrofluorescin diacetate (H2DCFDA) for 30 min, and the fluorescence intensities were measured by flow cytometry (channel FL1). The lipids were extracted and were quantified according to the method reported previously (Yoon *et al.*, 2012; Wu *et al.*, 2019).

## Results

### Identifying HpDGAT-encoding genes via mRNA-seq analysis and gene cloning

**Fig. 1.**
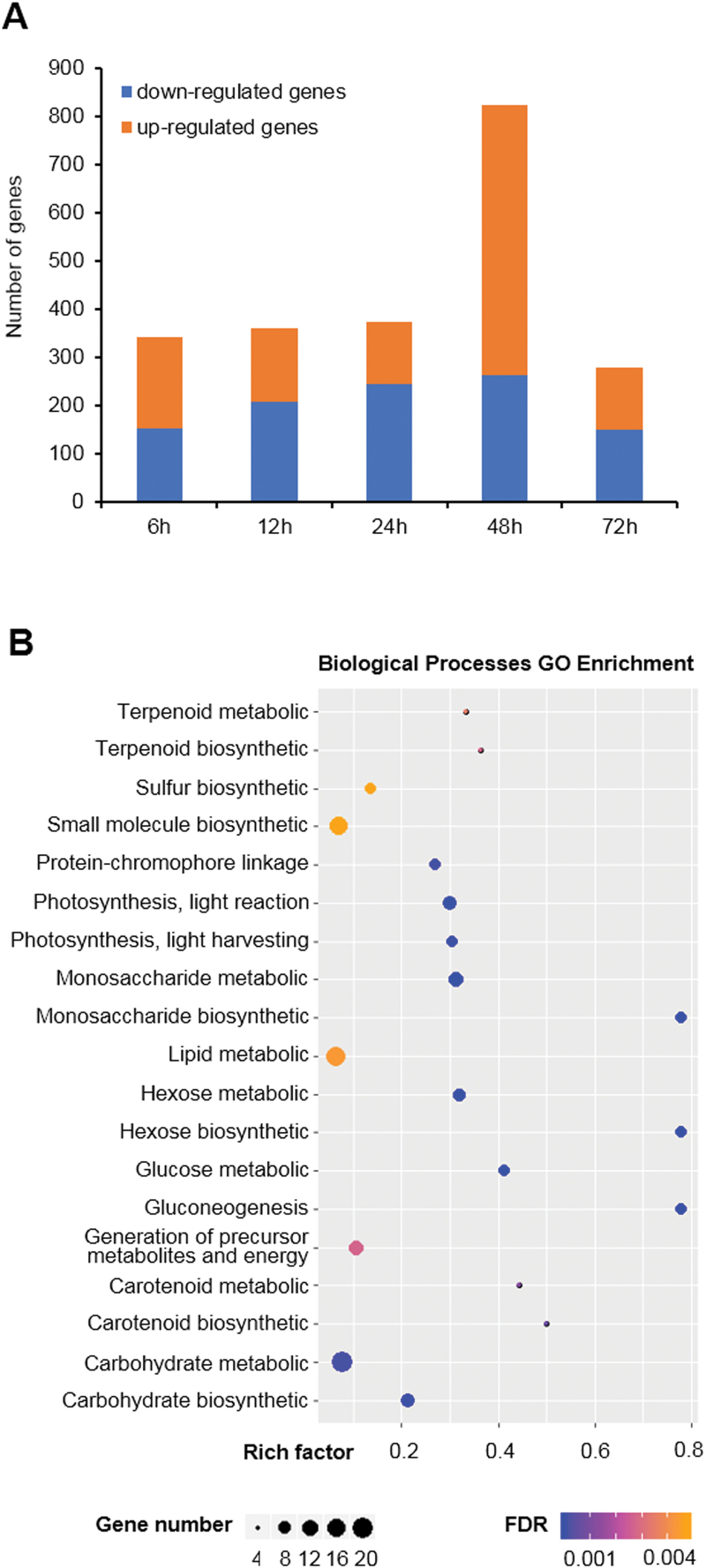
High-light-induced differentially expressed genes in *H. pluvialis*. (A) Number of differentially expressed genes calculated with fold change (high light/low light) ≥2 and FDR-corrected *P*-value ≤0.05. (B) Biological process GO enrichment of the differentially expressed genes at five time points.

To identify the DGAT-encoding genes, the global gene expression of *H. pluvialis* during different periods (6, 12, 24, 48, and 72 h) under HL and LL conditions was analyzed through mRNA-seq. Thirty high-quality transcript profiles were generated and showed high reproducibility (Spearman correlation >0.92) among the three biological replicates at each time point. A total of 12 752 genes were annotated from *de novo* assembly, including 1456 differentially expressed genes (Fig. 1A). The number of differentially expressed genes reached a maximum at 48 h after the start of exposure to HL, when it comprised 561 up-regulated and 263 down-regulated genes. GO and biological process analysis showed that differentially expressed genes were primarily enriched in gluconeogenesis, photosynthesis, lipid metabolic, and carotenoid biosynthetic processes over 72 h under HL, suggesting that these biological processes were susceptible to HL stress (Fig. 1B). Biosynthesis of monosaccharide, hexose, and gluconeogenesis process were the three most highly enriched categories among all the identified processes. The second most highly enriched processes were carotenoid biosynthesis and photosynthesis.

**Fig. 2.**
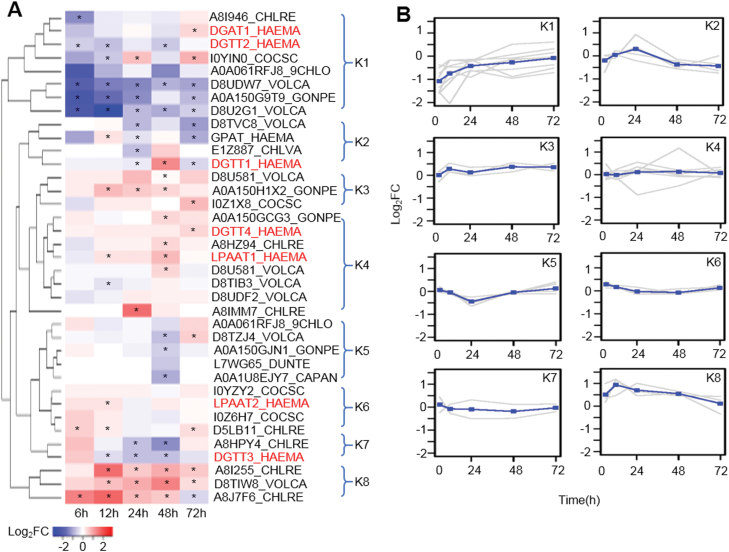
Putative acyltransferases in *H. pluvialis* and their expression pattern under high-light stress. (A) The 37 putative acyltransferase-encoding enzymes identified by mRNA-seq. *FDR<0.05, Benjamini–Hochberg adjustment. (B) Expression pattern of the enzymes grouped into the eight clusters shown in (A). The grey lines show the mean expression of each gene in each cluster. During the first 72 h, the mean log_2_ fold change (FC) of the genes in each cluster was plotted (lines with data points marked as squares).

A total of 37 putative acyltransferase-encoding genes were identified from the mRNA-seq dataset and were grouped into eight clusters based on their expression pattern in response to HL stress (Fig. 2A). Among these acyltransferase genes, one *DGAT1* and four *DGAT2* genes were identified, which is consistent with a previous study (Ma *et al.*, 2018). In this study, the *HpDGAT2* genes were named *HpDGTT1*, *HpDGTT2*, *HpDGTT3*, and *HpDGTT4*, according to the nomenclature system of *C. reinhardtii DGAT2*. As shown in Fig. 2, *HpDGAT1* and *HpDGTT2* were grouped together in cluster K1 and showed an initial suppression followed by an increase in transcript level under HL stress (Fig. 2B). The expression of *HpDGAT1* increased significantly (FDR<0.05) at 72 h, whereas the expression of *HpDGTT2* was suppressed under HL stress. *HpDGTT1* was distinct to cluster K2 and showed a fluctuating expression pattern, which increased significantly only at 48 h. *HpDGTT3* was grouped into cluster K7 and showed a transient increase followed by a significant decrease in transcript level. *HpDGTT4* was in cluster K4 and showed a slight but significant increase in transcript level at 72 h.

**Fig. 3.**
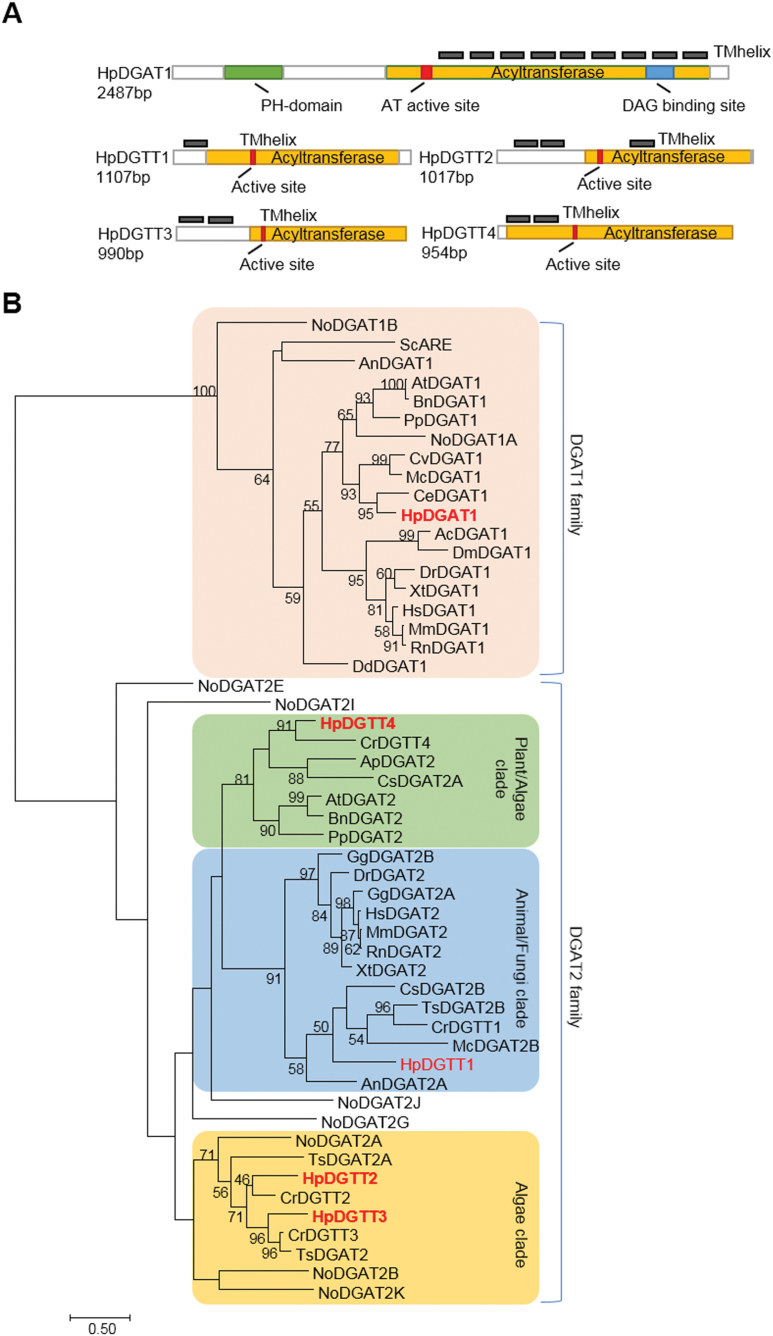
Cloning of *HpDGAT* genes and functional study of these genes in the *S. cerevisiae* H1246 system. (A) Coding sequences encoding protein structures. TM, transmembrane. (B) Phylogenetic tree of DGAT1s and DGAT2s. Ac, *Apis cerana*; At, *Arabidopsis thaliana*; An, *Aspergillus niger*; Ap, *Auxenochlorella protothecoides*; Bn, *Brassica napus*; Ce, *Chlamydomonas eustigma*; Cr, *Chlamydomonas reinhardtii*; Cs, *Chlorella sorokiniana*; Cv, *Chlorella vulgaris*; Dr, *Danio rerio*; Dd, *Dictyostelium discoideum*; Dm, *Drosophila melanogaster*; Ds, *Dunaliella salina*; Gg, *Gallus gallus*; Hs, *Homo sapiens*; Mc, *Micractinium conductrix*; Mm, *Mus musculus*; No, *Nannochloropsis oceanica*; Pp, *Physcomitrella patens*; Rn, *Rattus norvegicus*; Sc, *Saccharomyces cerevisiae*; Ts, *Tetrabaena socialis*; Xt, *Xenopus tropicalis*. Detailed information on the homologs is listed in Supplementary Table S2.

The RNA-seq results were validated by RT–PCR. The expression of the five identified *HpDGAT* genes at 12 h and 24 h under HL stress was compared. The *R*^2^ value was greater than 0.8 in the plot (Supplementary Fig. S1), indicating that the RNA-seq results were of good quality. The full-length cDNAs of the five *HpDGAT* genes were cloned from the cDNA library of *H. pluvialis* based on the gene sequence information obtained by RNA-seq. Their coding sequences encoding protein sequences are shown in Fig. 3A. The transmembrane domains of the five HpDGAT proteins predicted using three different algorithms are presented in Supplementary Table S3. In summary, except for HpDGTT1, the numbers of transmembrane domains predicted by TMHMM were less than or equal to those predicted by TMpred and HMMTOP, as the former algorithm ruled out the helixes with probability scores lower than 0.4. The other two algorithms, however, generated less stringent predictions, since those helixes with low scores were still predicted as transmembrane domains. The putative transmembrane domains consistent among the predictions of the three algorithms were adopted for further analysis. HpDGAT1 contains one pleckstrin homology (PH) domain and nine transmembrane domains. The alignment of HpDGAT1 with its homologs indicated that most DGAT1s contained isoleucine and leucine/isoleucine residues in the active sites, whereas HpDGTA1contained two alanine residues instead. The acyl acceptor binding site of HpDGAT1 was an alanine residue instead of the leucine, methionine, and cysteine residues common in other DGAT1s (Supplementary Fig. S2A). For HpDGAT2s, DGTT1 and DGTT2 contain one and three transmembrane domains, respectively, and DGTT3 and DGTT4 both contain two transmembrane domains. Like their homologs, the four HpDGAT2s contained the consensus YFP motif and activity-related HPHG motif (Wagner *et al.*, 2010) (Supplementary Fig. S2B). Meanwhile, three additional highly conserved domains were also found to be present in the DGAT2 family, including FxxPxxRxxxxxxG (PR block), GGxxE (GGE block), and RxGFxxxA-VPxxxFG (RGFA-VPFG block) (Supplementary Fig. S2B).

Phylogenetic analysis of protein sequences showed that HpDGTT1 was grouped with the DGAT2s of animals/fungi, while HpDGTT4 was closely related to the DGAT2s of plants (Fig. 3B). A separate cluster from the animal/fungi and plant DGAT2s, formed by HpDGTT2 and HpDGTT3 along with their homologs from *C. reinhardtii*, was designated as an algal lineage, which was located in the basal placement of the DGAT2 clade (Fig. 3B).

### Functional analysis of HpDGATs

**Fig. 4.**
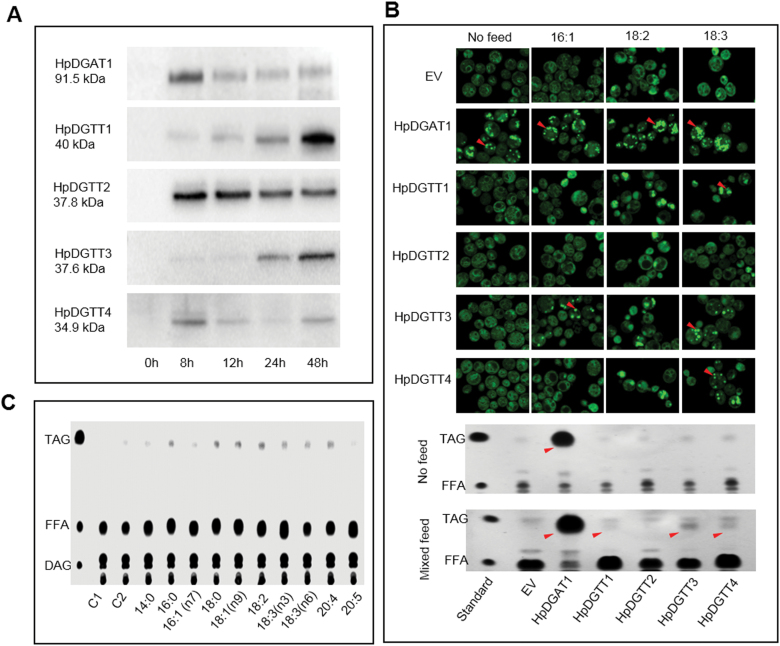
Functional study of *HpDGAT* genes in the *S. cerevisiae* H1246 system. (A) Recombinant protein expression in yeast microsomes after induction for up to 48 h. (B) Lipid bodies formed (upper panels) and TAG produced (lower panels) in *S. cerevisiae HpDGAT* transformants fed with or without fatty acids. FFA, free fatty acid. The upper panels show fluorescence staining with BODIPY 493/503 at a final concentration of 10μM. Arrowheads indicate individual lipid bodies. (C) Preference for acyl donors of HpDGAT1.

HpDGATs were overexpressed in the TAG synthesis-deficient *S. cerevisiae* H1246 (Tetra Mut. relevant genotype: *MAT*α *are1*-Δ::*HIS3 are2*-Δ::*LEU2 dga1*-Δ::*KanMX4 Iro1*-Δ::*TRP1 ADE2 ura3* TAG^−^ SE^−^) to investigate whether they can act as DGAT to catalyze TAG biosynthesis. As shown in Fig. 4A, the recombinant proteins resulting from expression of the five *HpDGAT* genes were accumulated in yeast cells after 8 h of induction. However, LBs were detected only in the transformants that overexpressed HpDGAT1 (Fig. 4B, lane “No feed”) after 24 h of induction, indicating that HpDGAT1 can function as a TAG synthase in the heterologous system. Feeding the transformants with exogenous C16:1, C18:2, and C18:3(n-3) fatty acids, which are minor fatty acids in this yeast species, rescued the TAG synthesis deficiency in *S. cerevisiae* H1246 harboring *HpDGTT3* and *HpDGTT4* (Fig. 4B). Although no typical LBs that could be stained with BODIPY were observed, the intracellular fluorescent signal was intensified in the *HpDGTT1* transformants compared with the strain transformed with empty vector (EV). The TLC analysis confirmed that the transformants harboring *HpDGAT1* and *HpDGAT2*s, except for *HpDGTT2*, produced TAG when they were fed with the mixture of exogenous fatty acids (Fig. 4B).

An *in vitro* enzymatic assay was performed with yeast microsomes containing the recombinant HpDGATs. A mixture of C16:1-C16:1 and C18:1-C18:1 DAGs was used as acyl acceptors, and 10 acyl-CoA species were used as acyl donors. HpDGAT1 showed enzymatic activities with a preference for C16:0, C18:0, C18:1(n-9), C18:2, and C20:4 acyl-CoAs (Fig. 4C). It could also utilize C18:3(n-3 and n-6) as a substrate, but with less selectivity. We failed to detect any TAG production with the four crude HpDGAT2 enzyme extracts, despite the fact that three of them showed DGAT activity *in vivo*. It is likely that the commercial acyl acceptors used in the assay were not suitable for HpDGAT2s, or that the protein activity in the microsomes was too weak to be detected *in vitro*. Taking the results together, we conclude that HpDGTT2 did not exhibit DGAT activity in either the *in vivo* or the *in vitro* analysis systems available.

### HpDGTT2 functions as a LPAAT

**Fig. 5.**
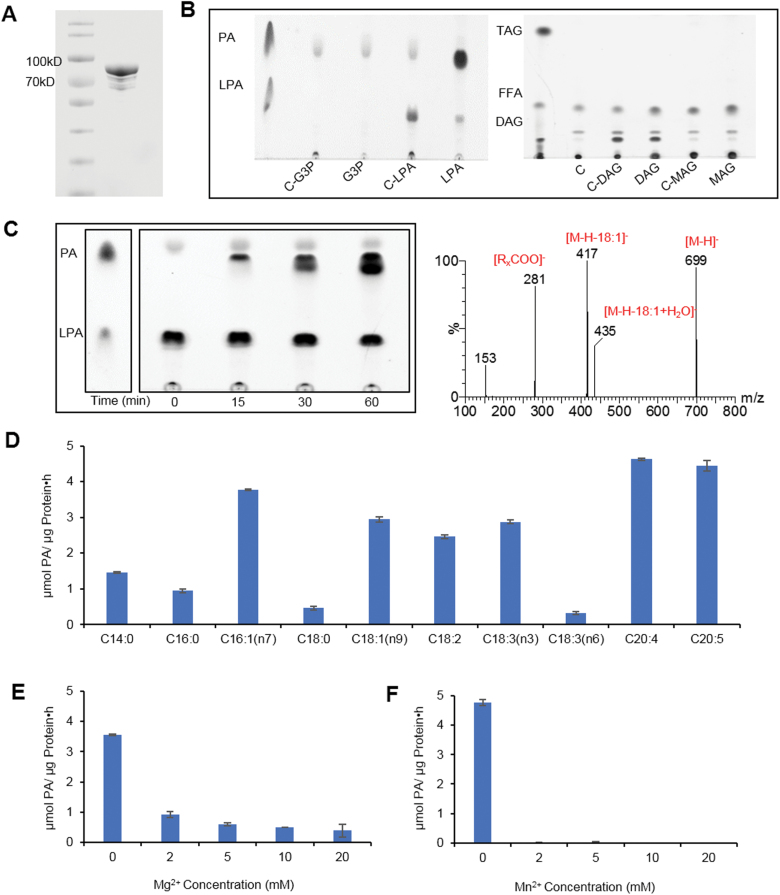
Functional characterization of recombinant DGTT2 protein in *in vitro* assays. (A) Expression of recombinant DGTT2. (B) Screen of acyl acceptor preferences. (C) Optimization of incubation time (left panel) and identification of the product with LC-MS (right panel). (D) Preference for acyl acceptors. (E) Effect of Mg^2+^ on the enzymatic activity. (F) Effect of Mn^2+^ on the enzymatic activity. Data are expressed as mean ±SD (*n*=2).

To decode the function of HpDGTT2, we obtained the recombinant HpDGTT2 in soluble form via fusion expression with a maltose-binding protein (MBP) tag. The solubility of HpDGTT2 with three putative transmembrane domains was dramatically increased by the MPB tag, enabling it to be purified for functional characterization (Fig. 5A). The enzymatic activity of HpDGTT2 was tested by using a variety of acyl acceptors, including G-3-P, LPA, DAG, and MAG. Among them, only LPA could be utilized by HpDGTT2 to produce a compound suspected to be PA, as indicated by TLC analysis (Fig. 5B). When C18:1-LPA and C18:1 acyl-CoA were incubated with HpDGTT2, the suspected PA product gradually accumulated over time (Fig. 5C). The identity of the product was confirmed by using the daughter scan mode of mass spectrometric analysis. The products with *m*/*z* of 699, 281, and 417 suggested a mass ion of C18:1/C18:1-PA, which yielded the fragment ions of C18:1 fatty acid ion and C18:1/C18:1-PA losing a C18:1 fatty acid moiety along with a proton, respectively (Fig. 5C). These results indicated that the synthesized product in the enzymatic assay with the purified HpDGTT2 was C18:1/C18:1-PA.

A substrate selectivity assay showed that HpDGTT2 can use a broad spectrum of acyl-CoAs but with a preference for unsaturated acyl-CoAs, such as C16:1(n-7), C18:3(n-3), C20:4, and C20:5-CoA (Fig. 5D). The LPAAT activity of HpDGAT was independent of Mg^2+^ and Mn^2+^, and in fact their presence could inhibit the enzymatic activity (Fig. 5E, F).

**Fig. 6.**
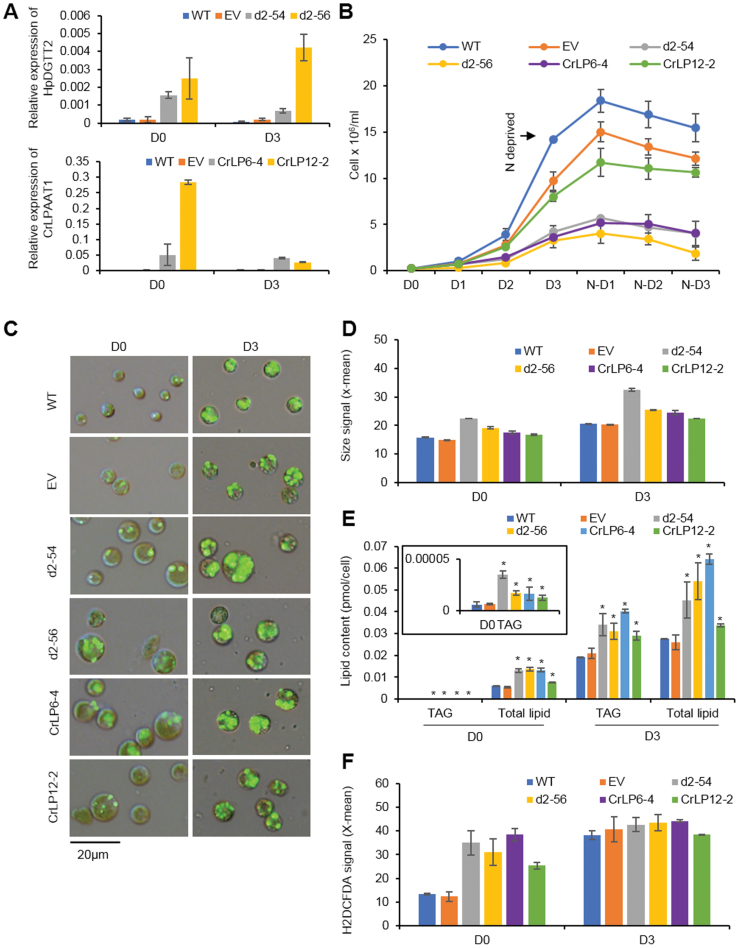
Phenotypes of *C. reinhardtii* CC-400 cw15 transformants overexpressing *HpDGTT2* and *CrLPAAT1* under nitrogen repletion and after 3 d of nitrogen deprivation. (A) Expression of *HpDGTT2* and *CrLPAAT1* in the *C. reinhardtii* transformants. (B) Growth of the transformants. (C) Cell phenotype of the transformants and lipid bodies formed within the cells. Fluorescence staining with BODIPY 493/503 at a final concentration of 10 μM. (D) Flow cytometry signal of forward scatter (size). (E) TAG and total lipid content of the transformants quantified by GC-MS. (F) Intracellular ROS level determined by H2DCFDA staining (final concentration of 10 μM). Data are expressed as mean ±SD, *n*=4 for (A) and (E), *n*=2 for (B), (D), and (F). *P<0.05 (Student’s *t*-test). WT, wild type; EV, empty vector transformant; d2, *HpDGTT2* transformant; CrLP, *CrLPAAT1* transformant; D0, day 0 after nitrogen deprivation (i.e. nitrogen repletion); D3, day 3 after nitrogen deprivation.

To further verify the function of HpDGTT2 as a LPAAT, we overexpressed *HpDGTT2* in *C. reinhardtii* strain CC-400 cw15. The phenotypes of the transgenic strains were compared with those of the reference strains with overexpression of *CrLPAAT1*, the authentic plastidial LPAAT-encoding gene from *C. reinhardtii* (Yamaoka *et al.*, 2016). The expression of *CrLPAAT1* and *HpDGTT2* in the transformants was confirmed by RT–qPCR (Fig. 6A). As shown in Fig. 6B, the growth of both *HpDGTT2* and *CrLPAAT1* transformants was significantly retarded compared with that of wild-type (WT) and EV transformed cells. Microscopic observation and flow cytometry analysis revealed that the cells of the *HpDGTT2* and *CrLPAAT1* transgenic strains were larger than those of the WT and EV controls (Fig. 6C, D), and both accumulated more TAGs under nitrogen-replete conditions (Fig. 6C).

The cellular contents of total lipids and TAGs were determined for the transgenic strains, WT, and EV through gas chromatography-mass spectrometry (GC-MS) analysis. As shown in Fig. 6E, the TAG contents of two *HpDGTT2* transgenic strains were 3.5×10^–5^ and 1.7×10^–5^ pmol cell^−1^, respectively, significantly (*P*<0.05) higher than those of WT and EV under nitrogen-replete conditions. Similarly, the TAG contents were significantly elevated in the *CrLPAAT2* transgenic strains. The cellular contents of total lipids were significantly increased in both *HpDGTT2* and *CrLPAAT1* transgenic strains as well, but to a lesser extent than the increase in TAG content, indicating that overexpression of both *HpDGTT2* and *CrLPAAT1* mainly contributed to TAG accumulation under nitrogen-replete conditions. Under nitrogen-depleted conditions, however, the increases in total lipids were more pronounced in the transgenic strains than the increases in TAG (Fig. 6E).

In addition, the intracellular oxidative status was determined by H2DCFDA staining. The results suggested that the ROS level in both *HpDGTT2* and *CrLPAAT1* transformants were higher than those in the WT and EV controls under nitrogen-replete conditions, suggesting that the transformants were subject to oxidative stress under normal conditions (Fig. 6F). The ROS level in both the controls and transformants increased in response to nitrogen deficiency. However, the ROS levels of the WT/EV controls and the transformants were almost identical under nitrogen deficiency, indicating that the transformants were less susceptible to nutrient stresses than the controls.

In summary, the transgenic *C. reinhardtii* strains overexpressing *HpDGTT2* and *CrLPAAT1* showed similar phenotypes, including enlarged cell size, retarded cellular growth, enhanced TAG biosynthesis, and higher intercellular ROS levels, providing further evidence for the *in vivo* function of HpDGTT2 as a LPAAT.

## Discussion

Functional divergences within the *DGAT2* gene family have been observed in many microalgae, such as *C. reinhardtii*, *C. zofingiensis*, and *N. oceanica*, mainly reflected by their different TAG synthase activities, substrate preferences, or subcellular localizations (Liu *et al.*, 2016; Xin *et al.*, 2017; Mao *et al.*, 2019; Xin *et al.*, 2019). These DGATs are believed to play overlapping but non-redundant roles in microalgal TAG biosynthesis. By contrast, a small number of putative DGATs from microalgae fail to show any expected activity in the well-established function verification systems, including complementation of *S. cerevisiae* H1246 and enzymatic assay with DAG and acyl-CoA as the substrates. Such a phenomenon is ubiquitous in microalgae harboring multiple copies of *DGAT2* genes, and has been reported in *C. reinhardtii*, *N. oceanica*, and *C. zofingiensis* (Liu *et al.*, 2016; Xin *et al.*, 2017; Mao *et al.*, 2019; Xin *et al.*, 2019). In this study, we decoded the functional divergence among five DGATs of the green microalga *H. pluvialis*, and unveiled a novel function of one isoform as a LPAAT.

*H. pluvialis* possesses four copies of DGAT2 proteins, which belong to the clades of DGATs of animals (i.e. HpDGTT1), eukaryotic algae (i.e. HpDGTT2 and HpDGTT3), and plants/green algae (i.e. HpDGTT4) (Fig. 3B). Among the four DGAT2 isoforms, HpDGTT1, HpDGTT3, and HpDGTT4 complemented TAG biosynthesis in *S. cerevisiae* H1246 with the addition of exogenous unsaturated fatty acids (Fig. 4B), albeit their enzymatic activities were too low to be detected *in vitro*. By contrast, and distinct from these typical DGAT2s, HpDGTT2 was found to play a role as a LPAAT.

With the evolution of enzymes, their catalytic activity and substrate selectivity tend to be specific. Although “moonlighting” functions and catalytic promiscuity have been observed with the functional release of more enzymes (Copley, 2003; Khersonsky *et al.*, 2006), the primary activity of acyltransferases is usually conserved within a given superfamily. For instance, DGAT2-related multifunctional acyltransferases of *Tetrahymena thermophila* are capable of esterifying different acyl acceptors, including fatty alcohols, diols, DAGs, and isoprenols, with acyl-CoA thioesters (Biester *et al.*, 2012). A multifunctional *O*-acyltransferase of the DGAT2/MGAT gene family that is highly expressed in human skin exhibits MGAT, wax synthase, and acyl-CoA:retinol acyltransferase activities (Yen *et al.*, 2005). Although these enzymes are multifunctional, they primarily act as DGATs. The catalytic promiscuity of these enzymes is probably a result of the substrate analogues used for the enzymatic assays in studies to characterize them. Here, for the first time, we report a DGAT2 isoform from *H. pluvialis* that has completely lost its DGAT function but acquired a novel function as a LPAAT.

**Fig. 7.**
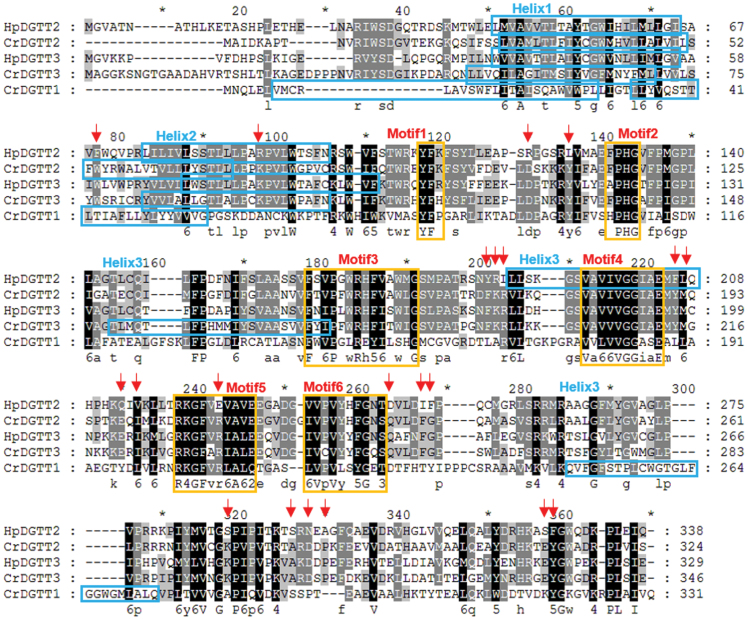
In-depth alignment of four DGAT2s from the algae clade and CrDGTT1. Predicted transmembrane helix domains and highly conserved motifs are indicated with boxes. Arrows indicate mutations in HpDGTT2.

A specific alignment of HpDGTT2 with the other three DGAT2s from the algal clade and one from the animal/fungi clade (i.e. CrDGTT1), which have been shown to be typical DGATs that can catalyze TAG formation in this work and a previous study (Liu *et al.*, 2016), revealed that more than 20 conserved AA residues in HpDGTT2 varied from the other three DGTT2s (Fig. 7, red arrows). These variant AAs that distinguish HpDGTT2 from the other DGAT2s were not located in the well-known conserved functional motifs, except for one basic AA, arginine (R), which was changed to the acidic AA glutamic acid (E) and located in the conserved domain RxGFxxA (Fig. 7, Motif 5). It is noteworthy that an extra predicted helical domain (from L188 to Q207) was present in HpDGTT2, delineated by two hydrophobic AA residues, FL, corresponding to two amphipathic AA, YM in the orthologs (Fig. 7, Helix 3). Additionally, three AA residues flanking this putative helical domain were YRI, varying from FKR in the orthologs. Notably, the highly conserved GGxxE motif (Fig. 7, Motif 4) was fully buried in the third helical domain in HpDGTT2. It is likely that the AA variation flanking the highly conserved motifs of the DGAT2 homologs determined the switch in the function of HpDGTT2. To further test this hypothesis, the crystal structure of HpDGTT2 needs to be resolved. The established methodology for the purification of such a highly hydrophobic protein paves a way for future crystallographic studies.

Phenotyping analysis of the transgenic strains suggested that overexpression of *HpDGTT2* and *CrLPAAT1* can lead to the arrest of algal cell division, which may result from the accumulation of PA, the product formed via the catalytic function of both of the enzymes encoded by these genes. PA has been identified as a multifunctional stress signaling lipid in plants, and the formation of PA can be triggered by various abiotic and biotic stresses, such as nutrient starvation, osmotic stress, pathogen attack, oxidative stress, as well as cold and freezing (Testerink and Munnik, 2005; Wang *et al.*, 2006). PA influences several physiological processes in plants, including ROS production and responses, activation of phosphatases and kinases, and hormone signaling (Wang *et al.*, 2006). In Arabidopsis, PA is suggested to increase the ROS level through increasing NADPH oxidase activity and activating the Rho-related small G protein GTPase-mediated pathway (Sang *et al.*, 2001; Park *et al.*, 2004). Consistent with these well-documented *in vivo* effects of PA, the ROS level was increased in the transformants harboring *HpDGTT2* and *CrLPAAT1*. In microalgae, TAG biosynthesis is thought to be subject to regulation by ROS, which is largely produced under environmental stresses such as nitrogen deficiency (Zhang *et al.*, 2013; Yilancioglu *et al.*, 2014). Thus, it can be deduced that the enhanced TAG accumulation is attributable not only to the increased LPAAT activity, but also to the ROS produced via the action of PA. On the other hand, ROS affects various cellular processes involved in cell cycle control through the phosphorylation and activation of numerous signal proteins (Boonstra and Post, 2004). Dependent on the exposure time and dose, ROS can cause either cell cycle arrest or proliferation (Boonstra and Post, 2004). We speculated that the increase in the cellular concentration of PA synthesized by HpDGTT2 or CrLPAAT1 may mimic stress signals, causing an increase in the intracellular ROS level, which led to both cell cycle arrest and the accumulation of TAG under nitrogen repletion observed in this study (Fig. 6). However, further evidence is needed to support this hypothesis.

Our results also suggested the HpDGAT1 was the major TAG synthetase in *H. pluvialis.* Among the four DGATs that can complement TAG biosynthesis in *S. cerevisiae* H1246, HpDGAT1 showed the highest activity, as indicated by the largest amounts of TAGs produced in the transformants (Fig. 4B). In addition, the recombinant HpDGAT1 was the only DGAT to exhibit active enzymatic function *in vitro* (Fig. 4C). Similar results have been observed in *N. oceanica* and *C. zofingiensis* (Wei *et al.*, 2017; Mao *et al.*, 2019): DGAT1A contributed ~25% of the TAG content under nitrogen deficiency in *N. oceanica* (Wei *et al.*, 2017), while overexpression of DGAT1A of *C. zofingiensis* increased the number and size of LBs in *S. cerevisiae* H1246 to the greatest extent, and contributed more than the other DGATs to LB formation in algal cells under nitrogen-deficiency stress (Mao *et al.*, 2019).

This study revealed unanticipated functional divergence of the DGAT family, and provides a framework to decrypt the structure–function relationship of both the conventional membrane-bound DGATs and those with novel functions yet to be identified. Such a framework can be leveraged for a broad spectrum of biotechnical applications.

## Supplementary data

The following supplementary data are available at *JXB* online.

Fig. S1. Validation of RNA-seq results with RT–qPCR.

Fig. S2. Alignments of DGAT1 and DGAT2 with homologs.

Table S1. List of primers used in this study.

Table S2. Information on DGAT homologs blasted with HpDGAT1 and four HpDGTTs.

Table S3. The transmembrane domain HpDGATs predicted by TMHMM, TMpred, and HMMTOP.

eraa451_suppl_Supplementary_File

## Data Availability

Sequence data from this article can be found in the GenBank data libraries (https://www.ncbi.nlm.nih.gov/genbank/) under accession numbers MN561784 (HpDGAT1), MN561785 (HpDGTT1), MN561786 (HpDGTT2), MN561787 (HpDGTT3), and MN561788 (HpDGTT4). Transcriptome data are available at NCBI Sequence Read Archive (https://www.ncbi.nlm.nih.gov/sra) with accession number PRJNA577112. The data supporting the findings of this study are available from the corresponding author, Danxiang Han, upon request.
