# Supplementary material for: Functional divergence of diacylglycerol acyltransferases in the unicellular green alga Haematococcus pluvialis
Source: J Exp Bot. 2020 Oct 1;72(2):510–24. doi: 10.1093/jxb/eraa451 (PMC7853605; doi:10.1093/jxb/eraa451)
Supplement: eraa451_suppl_Supplementary_File [file eraa451_suppl_supplementary_file.pdf]

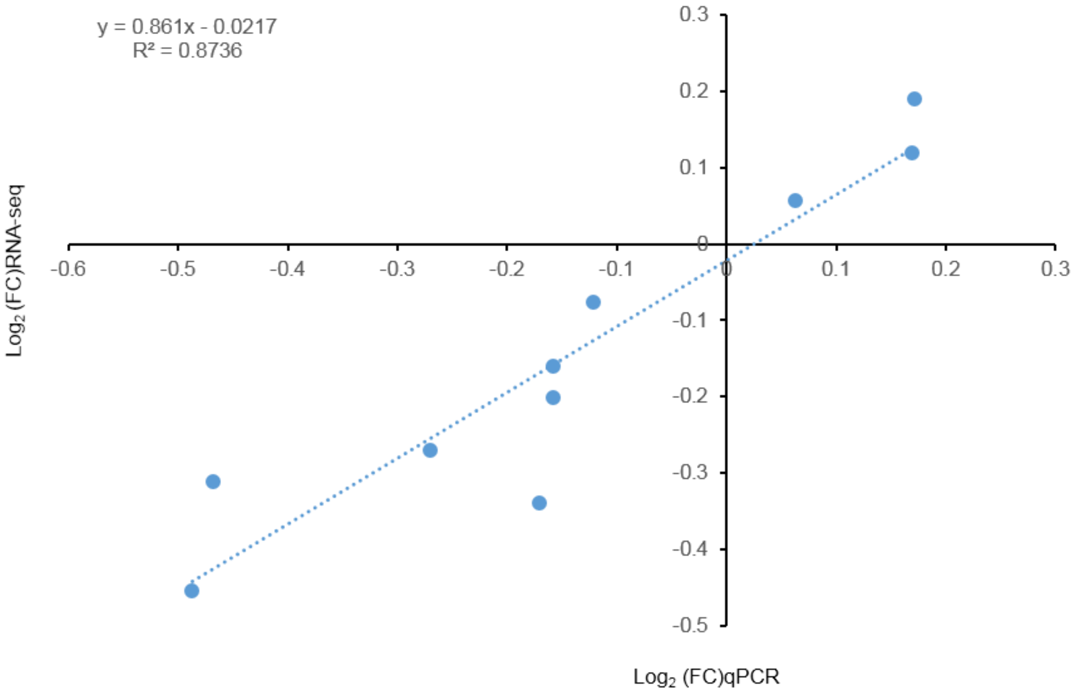

Fig. S1: Validation of RNA-seq result with qPCR. The expression of five HpDGAT genes at 12 and 24 h under high light stress were compared with the log_2 FC form. qPCR was performed in four replicates, and the data applied in the plot were expressed as mean.

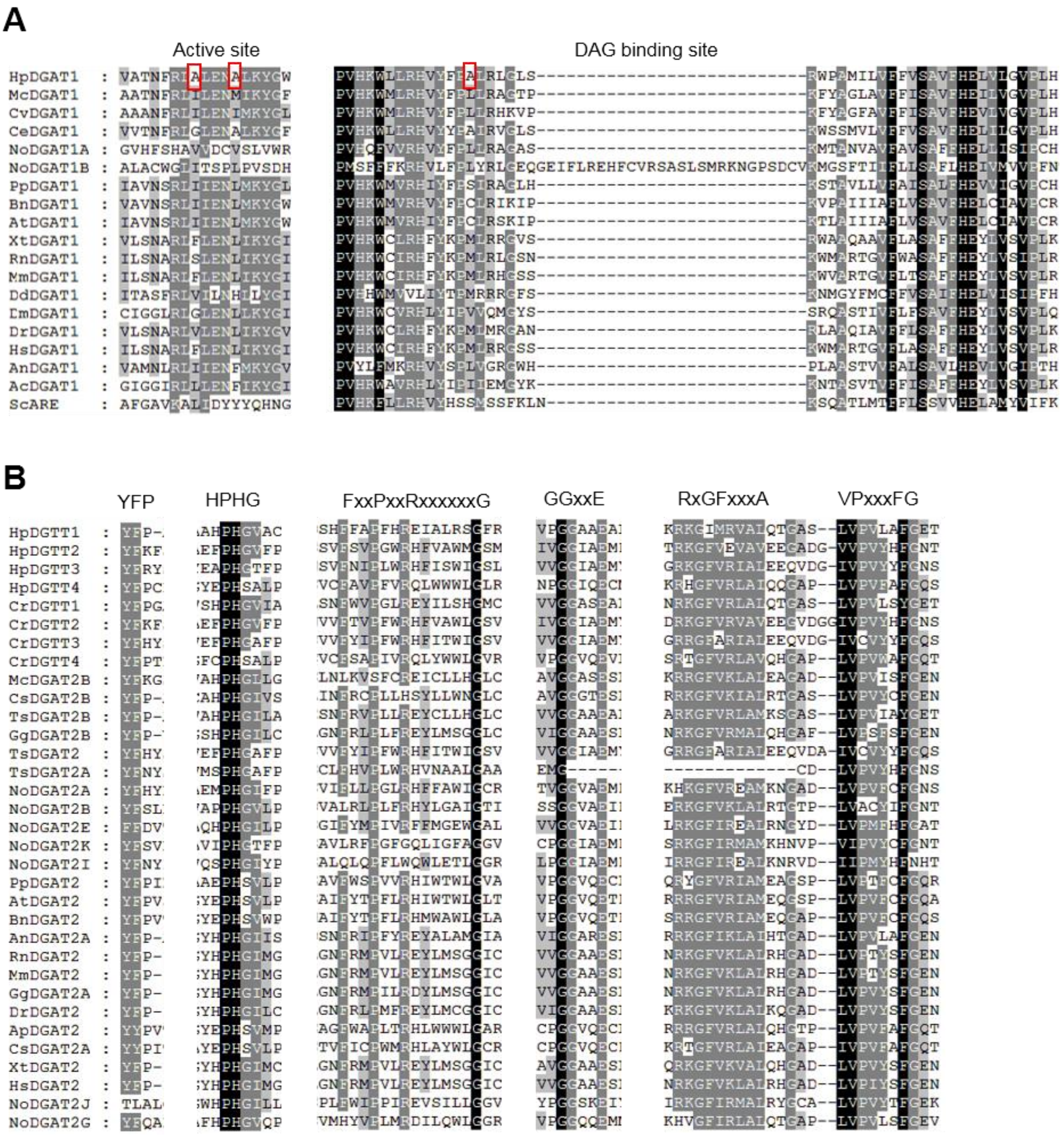

Fig. S2: Alignments of DGAT1 and DGAT2 with homologs. (A) DGAT1 Homologs. (B) DGAT2 homologs.

Table S1. Primer list

Item	Name	Sequence (5'-3')
qPCR validation of RNA-seq	DGAT1-3F	CTGCTGCGCCACGTCTACTT
	DGAT1-3R	GTTGCCAGCTTCGTCCCTGT
	DGTT1-4F	TGTCACAACCCGGGACATGC
	DGTT1-4R	CCGTGAATGAGGGGGATGGC
	DGTT2-4F	ACGCAAGGGGTTTGTGGAGG
	DGTT2-4R	GGGGATAGGTGACCCCGTGA
	DGTT3-4F	GAGCAGGTGGACGGCATTGT
	DGTT3-4R	GCGCTCAAACTCAGGGTCGT
	DGTT4-4F	CAGCTGTGGTGGTGGCTAGG
	DGTT4-4R	GGGGCGAAACCAGCTGTAGG
	18s rRNA-F	TGCCTAGTAAGCGCGAGTCA
	18s rRNA-R	CCCACCGCTAAAGTCAATCC
RACE	SmFs	GCAGTGGTATCAACGCAGAGTGGCCAT
	DGAT1-5'-R	GGGAGGTGCCTTGCAGGAGGAAGTAGC
	DGAT1-5'-nestR	CTGTGCATTTTGCGCCTGTAGCCGGAG
	DGTT1-5'-R	AGCCGCTGGCTTCAGTCAAGAAGTTGG
	DGTT1-5'-nestR	CCAGGTCTGCTGTCTTGTGCAGCTTG
	DGTT2-5'-R	ACGGGCATTCAATTCGTGCGTCTCCAG
	DGTT2-5'-nestR	CGTCGCATTCGTTGCGACACCCATTTG
	DGTT3-5'-R	ACCCATAGCTTGCAGAACGCAGTCCAC
	DGTT3-5'-nestR	GGCTTGGCTGGTAGCAGTAGTGTGCTC
	DGTT4-5'-R	TGCTAATCCAGCGAACCAGCCAGTCAG
	DGTT4-5'-nestR	GGGAGTGCGTGGCAAAGACAGTTGGT
	SmRs	TTCTAGAGGCCGAGGCGGCCGACAT
	DGAT1-3'-F	GGCTCAACATCCTTGCGGAGCTGCTG
	DGAT1-3'-nestF	GCAATGGAACCTGCCGGTGCACAAGT
	DGTT1-3'-F	TCACAACCCGGGACATGCGACCTGAT
	DGTT1-3'-nestF	AGCAACATGCCAACAGCTCATCCGCC
	DGTT2-3'-F	TGGTGCCCGTGTACCACTTTGGCAAC
	DGTT2-3'-nestF	ACGAAAACGAGTCGCAACGAGGCAGG

	DGTT3-3'-F	TGGTGGTTGGCGGCATCGCAGAAATG
	DGTT3-3'-nestF	ACTTCGGCAACAGCCAGGCCTTCAAC
	DGTT4-3'-F	AAGCCTGACTGGCTGGTTCGCTGGAT
	DGTT4-3'-nestF	CGCTTCATACACGACATGGCGGCGTT
pYES2-DGAT vectors	DGAT1-Y-F	cgc*ggatcc*ATGCGCATTATGCCAT
	DGAT1-Y-R	ctag*tctaga*GATGACAATGCCGGAGCTTGTG
	DGTT1-Y-F	cg*ggatcc*ATGGCTGGTGATACTG
	DGTT1-Y-R	gc*tctaga*CTGCACAAACTCCAGGCTC
	DGTT2-Y-F	cg*gaattc*ATGGGTGTCGCAACGAATGC
	DGTT2-Y-R	gc*tctaga*CTGGATCTCCAGGGGCTTGTC
	DGTT3-Y-F	cc*aagctt*ATGGGCGTTAAAAAGC
	DGTT3-Y-R	ccg*gaattc*CTCGATGCTCAG
	DGTT4-Y-F	cc*aagctt*ATGCCGGCGTTGC
	DGTT4-Y-R	tgc*tctaga*CAAGATGCGCAACTCG
pMAL-c5x vector	pMAL-Dg2-F	*ttgcggccgc*ATGATGGGTGTCGC
	pMAL-Dg2-R	*cggaattc*TCACTGGATCTCCAGGGGCT
pClamy-4 vector	P4-DGTT2-F	ccggaattcATGGGTGTCGCAACGAAT
	P4-DGTT2-R	gctctagaTCACTGGATCTCCAGGGGCT
	P4-LPAAT1-F	ccggaattcATGGCGCGTAAAAGCAGT
	P4-LPAAT1-R	gctctagaTCACTGCTCATCCGGCGC
qPCR in *C. reinhardtii* overexpression system	CBLP-F	GACCACCAACCCCATCATC
	CBLP-R	AGACGGTCACGGTGTTGAC
	DGTT2-4F	ACGCAAGGGGTTTGTGGAGG
	DGTT2-4R	GGGGATAGGTGACCCCGTGA
	CrLPAAT1-F	ACATCTACTCGCTGTTCCACCTG
	CrLPAAT1-R	CGGTCCACACGGTTTATCATCAC

Table S2. Information of DGAT homologs blasted with HpDGAT1 and four of HpDGAT2s.

Species name	Acronym	Score	E-value	accession NO
**HpDGAT**				
Dunaliella salina	Ds	625	0	QID75817
Micractinium conductrix	Mc	511	3.00E-169	PSC71143
Chlorella vulgaris	Cv	447	8.00E-148	ALP13863
Chlamydomonas eustigma	Ce	429	6.00E-134	GAX78660
Nannochloropsis oceanica DGAT1A	No	266	9.00E-79	ASL69957
Nannochloropsis oceanica DGAT1B	No	160	5.00E-39	ASL69958
Physcomitrella patens	Pp	389	1.00E-124	XP_024395838
Brassica napus	Bn	357	1.00E-112	NP_001303201
Arabidopsis thaliana	At	347	1.00E-108	NP_179535
Xenopus tropicalis	Xt	274	2.00E-81	XP_031759943
Rattus norvegicus	Rn	271	3.00E-80	NP_445889
Mus musculus	Mm	268	4.00E-79	NP_034176
Dictyostelium discoideum AX4	Dd	268	2.00E-77	XP_645633
Drosophila melanogaster	Dm	266	2.00E-77	AAL78365
Danio rerio	Dr	261	2.00E-76	NP_956024
Homo sapiens	Hs	259	7.00E-76	NP_036211
Aspergillus niger	An	247	8.00E-70	GAQ45059
Apis cerana	Ac	241	7.00E-69	XP_016909650
Saccharomyces cerevisiae YJM326 ARE2p	Sc	149	1.00E-35	AJT04763
**HpDGTT1**				
Chlamydomons reinhardtii DGTT1	Cr	212	3.00E-64	AGO32156
Micractinium conductrix DGAT2B	Mc	194	2.00E-56	PSC71349
Chlorella sorokiniana DGAT2B	Cs	211	1.00E-63	PRW45193
Tetrabaena socialis DGAT2B	Ts	202	2.00E-61	PNH12928
Xenopus tropicalis DGAT2	Xt	172	8.00E-49	NP_989372
Dictyostelium discoideum AX4 DGAT2	Dd	197	1.00E-58	XP_635762
Danio rerio DGAT2	Dr	180	1.00E-51	NP_001025367
Aspergillus niger ATCC 13496 DGAT2A	An	192	1.00E-55	RDH24132
Gallus gallus DGAT2B	Gg	185	2.00E-53	AOV63266

**HpDGTT2**				
Chlamydomons reinhardtii DGTT2	Cr	360	7.00E-123	AGO32157
Chlamydomons reinhardtii DGTT3	Cr	330	8.00E-111	AGO32158
Tetrabaena socialis DGAT2	Ts	336	7.00E-113	PNH06113
Tetrabaena socialis DGAT2A	Ts	168	5.00E-46	PNH04213
Nannochloropsis oceanica strain IMET1 2A	No	187	8.00E-55	ATB53137
Nannochloropsis oceanica strain IMET1 2B	No	115	1.00E-27	ATB53138
Nannochloropsis oceanica strain IMET1 2E	No	100	2.00E-22	ATB53141
Nannochloropsis oceanica strain IMET1 2K	No	98.6	2.00E-21	ATB53136
Nannochloropsis oceanica strain IMET1 2I	No	94	2.00E-19	ATB53145
Physcomitrella patens DGAT2	Pp	127	2.00E-32	XP_024360450
Arabidopsis thaliana DGAT2	At	125	2.00E-31	NP_566952
Brassica napus DGAT2	Bn	124	5.00E-31	XP_013745595
Xenopus tropicalis DGAT2	Xt	94.4	7.00E-20	NP_989372
Danio rerio DGAT2	Dr	92.4	3.00E-19	NP_001025367
Gallus gallus DGAT2A	Gg	90.5	1.00E-18	XP_419374
Aspergillus niger ATCC 13496 DGAT2A	An	97.1	1.00E-20	RDH24132
Rattus norvegicus DGAT2	Rn	90.9	1.00E-18	NP_001012345
Mus musculus DGAT2	Mm	90.1	9.00E-19	EDL16369
**HpDGTT3**				
Chlamydomonas reinhardtii CrDGTT3	Cr	394	8.00E-136	AGO32158
Chlamydomonas reinhardtii CrDGTT2	Cr	328	2.00E-110	AGO32157
Tetrabaena socialis DGAT2	Ts	386	7.00E-133	PNH06113
Tetrabaena socialis DGAT2A	Ts	186	1.00E-52	PNH04213
Nannochloropsis oceanica strain IMET1 2A	No	198	4.00E-59	ATB53137
Nannochloropsis oceanica strain IMET1 2B	No	114	4.00E-27	ATB53138
Nannochloropsis oceanica	No	111	1.00E-25	ATB53145

strain IMET1 2I				
Nannochloropsis oceanica strain IMET1 2K	No	104	1.00E-23	ATB53136
Nannochloropsis oceanica strain IMET1 2E	No	102	4.00E-23	ATB53141
Arabidopsis thaliana DGAT2	At	114	1.00E-27	NP_566952
*Brassica napus*DGAT2	Bn	112	1.00E-26	XP_013745595
Physcomitrella patens DGAT2	Pp	109	1.00E-25	XP_024360450
Xenopus tropicalis DGAT2	Xt	95.5	2.00E-20	NP_989372
Gallus gallus DGAT2A	Gg	97.4	5.00E-21	XP_419374
Danio rerio DGAT2	Dr	96.3	1.00E-20	NP_001025367
Chlorella sorokiniana DGAT2B	Cs	80.5	4.00E-15	PRW45193
Mus musculus DGAT2	Mm	79.7	4.00E-15	EDL16369
**HpDGTT4**				
Chlamydomonas reinhardtii CrDGTT4	Cr	295	1.00E-97	AGO32159
Arabidopsis thaliana DGAT2	At	228	2.00E-71	NP_566952
Brassica napus DGAT2	Bn	223	3.00E-69	XP_013745595
Physcomitrella patens DGAT2	Pp	217	3.00E-67	XP_024360450
Auxenochlorella protothecoides DGAT2	Ap	219	9.00E-68	XP_011401553
Chlorella sorokiniana DGAT2A	Cs	210	3.00E-64	PRW21030
Xenopus tropicalis DGAT2	Xt	125	1.00E-31	NP_989372
Homo sapiens DGAT2	Hs	123	1.00E-30	NP_001240820
Danio rerio DGAT2	Dr	124	5.00E-31	NP_001025367
Mus musculus DGAT2	Mm	119	1.00E-29	EDL16369
Rattus norvegicus DGAT2	Rn	122	6.00E-30	NP_001012345
Gallus gallus DGAT2A	Gg	118	7.00E-29	XP_419374
Nannochloropsis oceanica strain IMET1 2A	No	110	6.00E-26	ATB53137
Nannochloropsis oceanica strain IMET1 2J	No	112	7.00E-26	ATB53146

Table S3. The transmembrane domain HpDGATs predicted by TMHMM, TMpred and HMMTOP.

Name	Length(AA)	TMHMM	TMpred	HMMTOP
HpDGAT1	828	9	9	9
HpDGTT1	368	2	3	1
HpDGTT2	338	3	4	3
HpDGTT3	329	2	5	5
HpDGTT4	317	2	5	5
